# Increased Levels of Antinutritional and/or Defense Proteins Reduced the Protein Quality of a Disease-Resistant Soybean Cultivar

**DOI:** 10.3390/nu7075269

**Published:** 2015-07-22

**Authors:** Daniele O. B. Sousa, Ana F. U. Carvalho, José Tadeu A. Oliveira, Davi F. Farias, Ivan Castelar, Henrique P. Oliveira, Ilka M. Vasconcelos

**Affiliations:** 1 Department of Biochemistry and Molecular Biology, Federal University of Ceará, Campus do Pici, Fortaleza 604440-900, CE, Brazil; E-Mails: danisilas@yahoo.com.br (D.O.B.S.); jtaolive@ufc.br (J.T.A.O.); daviffarias@yahoo.com.br (D.F.F.); henriquebio@gmail.com (H.P.O.); 2 Department of Biology, Federal University of Ceará, Campus do Pici, Fortaleza 60440-900, CE, Brazil; 3 Department of Finance, Federal University of Ceará, Campus Benfica, 60440-970, Fortaleza 60020-180, CE, Brazil; E-Mail: lume1250@yahoo.com.br

**Keywords:** Seridó-RCH, unintended traits, conventional breeding, nutritional value, plant protein

## Abstract

The biochemical and nutritional attributes of two soybean (*Glycine max* (L.) Merr.) cultivars, one susceptible (Seridó) and the other resistant (Seridó-RCH) to stem canker, were examined to assess whether the resistance to pathogens was related to levels of antinutritional and/or defense proteins in the plant and subsequently affected the nutritional quality. Lectin, urease, trypsin inhibitor, peroxidase and chitinase activities were higher in the resistant cultivar. Growing rats were fed with isocaloric and isoproteic diets prepared with defatted raw soybean meals. Those on the Seridó-RCH diet showed the worst performance in terms of protein quality indicators. Based on regression analysis, lectin, trypsin inhibitor, peroxidase and chitinase appear to be involved in the resistance trait but also in the poorer nutritional quality of Seridó-RCH. Thus, the development of cultivars for disease resistance may lead to higher concentrations of antinutritional compounds, affecting the quality of soybean seeds. Further research that includes the assessment of more cultivars/genotypes is needed.

## 1. Introduction

Natural genetic variation within species and among related species has been one of the main sources of diversity for crop improvement [1]. For centuries, conventional plant breeding programs have produced new traits, higher yields and improved quality. However, little attention has been paid to metabolic changes occurring in successive generations. This issue has gained importance only recently in the context of safety assessment of genetically modified (GM) crops [2]. This issue is also of great significance because several studies have reported wide variation in the chemical and nutritional composition of genetically close cultivars of important legume seeds, such as soybeans, obtained by conventional plant breeding [3,4]. Nevertheless, little is known about the consequences of the observed differences resulting from plant breeding and the impact of the possible unintended effects on the nutritional value of crops.

The impairment of legumes’ nutritional value is attributed in part to the presence of different compounds classically known as toxic and/or antinutritional factors, which act as direct or indirect antagonists of nutrient availability [5]. For soybean seeds, trypsin inhibitor (SBTI) and lectin (SBA) are considered to be the major proteins responsible for poor nutritional value [6]. In fact, soybean varieties deficient in Kunitz protease inhibitor or in lectin have shown improved nutritional quality [7,8]. It has been suggested that other proteins, such as soybean toxins that are lethal to mice when administered by the intraperitoneal route and urease, might also contribute to the deleterious effects observed upon feeding soybeans to rats [4]. Of lesser significance are antinutritional factors that are non-protein in nature, such as goitrogens, tannins, phytoestrogens, flatus-producing oligosaccharides, phytate, and saponins [9]. In the present work, we focused only on protein antinutritional factors.

The defensive role of lectins and trypsin inhibitors against phytopathogens and insects is well known [10,11], but in contrast to the toxic/antinutritional effects, this role cannot be effective when the factors are present at very low levels, which in turn could depress plant performance in general. In addition, reduced levels can decrease plant performance in the field. Considering the scarcity of information on the correlation of plant pathogen resistance and nutritional value, there is a need for studies to ascertain whether the proteins possibly responsible for resistance interfere with the nutritional quality of the cultivar.

In the above context, the soybean cultivar Seridó-RCH was obtained through conventional plant breeding (backcrossing of the Seridó cultivar) by EMBRAPA (Brazilian Agricultural Research Corporation) in experimental fields located in Balsas, Maranhão, Brazil. The parental cultivar Seridó (BR 28) corresponds to the progeny-F6, identified by the name BR 83–9221, which originated from the crossing of Santa Rosa with BR 78–11202. The resulting seeds in F1 were harvested and used to produce F2 and successive crosses of segregating generations through planting and harvesting to the desired level of homozygosis, which was achieved by F6 inbreeding. Seridó (BR 28) possesses the resistance trait against frogeye leaf spot caused by *Cercospora sojina*, but it is susceptible to both stem canker and bacterial pustules caused by *Diaporthe phaseolorum* f.sp. *meridionalis* and *Xanthomonas axonopodis* pv. *glycines*, respectively. Seridó-RCH, also denominated BR 96–4909 or MA/BRS-165 cultivar, was obtained by backcrossing of BR 28 (Seridó) with the Embrapa 20 (Doko RC) cultivar for incorporation of resistance to stem canker. In fact, Seridó-RCH was shown to be resistant to stem canker and frogeye leaf spot and also to bacterial pustules. These are three major diseases for soybean culture [12]. Nevertheless, nothing is known about the composition or nutritional value of these cultivars and whether the acquired high resistance to pathogens is associated with certain unintended nutritional effects that could have emerged as a result of the process of backcrossing to obtain Seridó-RCH. This is possible for antinutritional factors, as a similar outcome was observed following the emergence of resistance to bacterial pustules when backcrossing was performed to obtain Seridó-RCH. Thus, this work aimed to perform a comparative study with emphasis on the protein quality of both Seridó and Seridó-RCH.

Different markers and approaches can be used to assess the nutritional quality of proteins, such as the Protein Digestibility-Corrected Amino Acid Score (PDCAAS) and the indispensable Amino Acid Score (AAS) [13]. However, animal feeding studies may provide additional useful information to complement safety and nutritional value assessments of food and feed, especially when unintended effects are suspected. Additionally, *in vivo* assays allow the analysis of crucial parameters related to protein quality, such as the balance between the amount of nitrogen retained and lost by animal tissues. Moreover, it is possible to observe systemic effects that can culminate with poor development or that can cause alterations in the vital organs of animals. Thus, in the current study, a traditional short-term feeding trial was organized in which we could measure several parameters, such as food intake, body weight gain, net protein utilization (NPU), protein digestibility and biological value. The rat was selected for the study because this species has been traditionally used to assess the safety of food. In addition, the levels of several classical antinutritional and defense proteins were measured, and the results were correlated to the nutritional responses of the feeding trial.

## 2. Experimental Section

### 2.1. Biological and Chemical Reagents

Soybean seeds of the Seridó and Seridó-RCH cultivars were developed and supplied by EMBRAPA (Experimental Field at Balsas, Maranhão, Brazil). The sowing was performed in soil (Oxisol) previously fertilized with NPK (2:20:20) at a dose of 400 kg/ha. Seeds (5 kg per cultivar) were obtained from three different plots. The plots consisted of 8 rows, each 8 m in length and equally spaced at 0.5 m apart, with a population density of 250,000 plants/ha maintained under continuous irrigation from planting until harvesting. Seeds were sowed in mid-November and harvested from March to April 2010, when the plants reached the R8 development stage and moisture content between 14% and 16%. After harvesting, the seeds were stored in sealed packages in a ventilated area free of fungi and rodents, and the temperature and humidity were maintained below 25 °C and 70%, respectively.

Male Wistar rats were obtained from outbred colonies maintained at the Federal University of Ceará (Fortaleza, Brazil). This institution has adopted Poiley’s system of breeding to avoid consanguinity and to preserve colony heterogeneity. The animals were maintained in an appropriate climatized environment, with controlled conditions of temperature (22 ± 2 °C), humidity (55%) and photoperiod (12 h of light/12 h of dark). Prior to the experiments, the animals were maintained with a pelletized diet and drinking water, both of which were sterilized. Rabbit blood was obtained from animals in colonies maintained at the same institution.

Egg whites (EWs) from chicken (Sigma E0500), β-glucuronidase (Sigma G0251), guaiacol (Sigma G5502), laminarin (Sigma L9634), l-BAPNA (Sigma B3133), *N*-acetyl-d-glucosamine (Sigma A8625), soybean trypsin inhibitor (Sigma T9128), trypsin (Sigma T6424) and urease (Sigma U0251) were purchased from Sigma-Aldrich Co. LLC (St. Louis, MO, USA). All other chemical reagents used in the experiments were of analytical grade.

### 2.2. Determination of Classical Antinutritional Proteins

Mature seeds were ground in a coffee grinder fitted with a 1-mm mesh screen, and the resulting flour was treated with petroleum ether (1:10, w/v) to extract lipids that could interfere with the protein determination. The solvent petroleum ether was evaporated to dryness under a gentle stream of air at room temperature (22 ± 3 °C). The defatted flour was extracted with 0.025 M Tris-HCl, pH 7.5 (1:5, w/v) [14]. The crude extract obtained was used for detection of the lectin trypsin inhibitor and urease activities. Lectin activity was assessed following a serial two-fold dilution of the samples [14]. The extracts were diluted with 0.15 M NaCl and mixed with a 2% suspension of rabbit erythrocytes prepared in 0.15 M NaCl. The degree of agglutination was monitored visually after the tubes had been left to stand at 37 °C for 30 min and after an additional 30 min at room temperature (22 ± 3 °C). The minimal protein concentration after serial dilution that still promoted agglutination visible to the naked eye was used to calculate the lectin equivalents. The lectin activity was expressed as grams of lectin equivalents per kilogram of soybean flour. Trypsin inhibitor activity was examined using trypsin and l-BAPNA as substrate. The activity was expressed as the amount (g) of trypsin inhibited per kilogram of soybean flour [15]. Urease assay was assessed using the procedure described by Kaplan, with minor modifications, and the urease activity was expressed as units of enzyme per kilogram of soybean flour [16]. For preparation of urease solution (50 μg/mL), glycerol was added to 0.2 M phosphate buffer, pH 6.5, containing 2% EDTA, at a ratio of 1:4 to preserve this solution for about three weeks at 4 °C. In addition, to obtain a standard curve, urea was used at 0.5 M instead of 0.3 M and prepared in the same buffer above.

### 2.3. Determination of Classical Plant Defense Proteins

Crude extracts (1:5, w/v) were prepared with 0.025 M Tris-HCl, pH 7.5, as described below and were used for the detection of chitinase, peroxidase and β-1,3-glucanase. The determination of peroxidase activity was performed using 0.02 M guaiacol and 0.06 M hydrogen peroxide as a donor and an acceptor of protons, respectively. An increase of one absorbance unit per minute at 480 nm was used as a unit of peroxidase activity (1 UAP) [17]. Chitinase activity determination in the crude extract was carried out by measuring the *N*-acetyl-d-glucosamine released due to hydrolytic enzyme action on colloidal chitin. Chitinase activity was expressed as nanokatal (nkat) per gram of seed flour (nkat/g) and was defined as the amount of enzyme that releases 1 nmol *N*-acetyl-d-glucosamine per second [18]. β-1,3-Glucanase activity was expressed in nkat per gram of seed flour (nkat/g) and was defined as the amount of enzyme necessary to catalyze the formation of 1 nmol of glucose equivalent per second [18].

### 2.4. Amino Acid Composition

Defatted soybean flour was hydrolyzed with 6 M HCl containing 10 g/L phenol at 110 °C for 22 h in sealed glass tubes under a N_2_ atmosphere. HCl and phenol were removed by evaporation, and the amino acid composition was established after chromatography on a Biochrom 20 system (Pharmacia). Tryptophan was determined according to the method described by Pinter-Szakács and Molnár-Perl [19]. All determinations were run in triplicate.

### 2.5. Diets

All soybean diets were prepared using seeds produced under strict quality standards. In addition, as the main concern of this work was to compare the nutritional quality of the seed proteins of two soybean cultivars, any type of processing (such as autoclaving or irradiation) that could cause changes in compounds, including anti-nutritive proteins, was not applied, except for the process of defatting the soybean flours. This step was performed to allow formulation of the 10% protein test diets with the same amount and source of lipids (maize oil) as in the control diet. Aflatoxin-free soybean (Seridó and Seridó-RCH) samples, confirmed by analysis carried out with a commercial competitive enzyme-linked immunosorbent assay (ELISA) kit [20], were ground in a hygienized blender and then refined using a coffee grinder (mesh size 1.0 mm). Powdered diets (1 kg) were formulated to contain the equivalent of 100 g protein per kilogram of diet (Table 1) in the form of EW protein or seed meal from cv. Seridó-RCH or Seridó. Diets containing the respective seed flours were supplemented with l-tryptophan and l-methionine based on the amino acid contents of the raw seeds to bring the amino acid content to the target requirements for rats [21]. Diet ingredients were mixed exhaustively in a nutrition laboratory in a flour homogenizer (SY-SM10 Spiral Mixer, Sunrry Machinery Technology Co., Ltd., Guangzhou, China) under adequate sanitary conditions and then analyzed for protein content to confirm the theoretical values. Before starting the feeding trial, the animals were submitted to a 5-day period of adaptation to the powdered diets. This protocol had been previously established in studies performed in metabolic cages suited for powdered diets (Nalgene Metabolic Cages, Buguggiate, Italy). A diet containing no protein (non-protein control, or NPC) was fed to a group of animals to allow determination of certain nutritional parameters.

**Table 1:** Composition (g/kg) of non-protein control (NPC), egg white (EW) and experimental diets.

Ingredient	NPC	EW	Seridó	Seridó-RCH
maize starch	555.6	422.4	409.4	399.6
cassava starch	111.1	111.1	100.0	100.0
glucose	166.7	166.7	150.0	150.0
maize oil	55.6	55.6	50.0	50.0
vitamin mix *^a^*	55.6	55.6	50.0	50.0
mineral mix *^b^*	55.6	55.6	50.0	50.0
EW	-	133.1	-	-
Seridó	-		187.6	-
Seridó-RCH	-		-	197.4
l-methionine *^c^*	-		2.3	2.3
l-tryptophan *^c^*	-		0.7	0.7
Energy *^d^* (kcal/g)	3.4	3.4	3.6	3.6

*^a^* Vitamin mix (g/kg diet): vitamin B_12_ (100%), 0.02; folic acid, 0.04; biotin (1%), 4.0; pyridoxine HCl, 0.04; thiamine HCl, 0.06; riboflavin (99%), 0.21; Ca-pantothenate (45%), 1.2; nicotinic acid, 4.0; inositol, 4.0; *p*-amino-benzoic acid, 12.0; choline chloride (50%), 24.0; maize starch, 950.43. *^b^* Mineral mix (g/kg diet): calcium citrate, 296.1; calcium carbonate (40%), 65.8; copper carbonate, 1.1; magnesium carbonate, 34.3; zinc carbonate, 0.48; ferric citrate, 9.1; magnesium chloride.6H_2_O, 5.82; sodium chloride, 74.0; potassium chloride, 119.5; monobasic calcium phosphate, 108.2; dibasic potassium phosphate, 210.1; sodium fluoride, 0.48; potassium iodate 0.1; magnesium sulfate, 75.4. *^c^* Diets containing raw seed flour of the distinct cultivars were supplemented with l-methionine and l-tryptophan according to their amino acid compositions. *^d^* The energy content was estimated by multiplying the percentages of crude protein, crude fat, and carbohydrates by their respective modified Atwater factors.

### 2.6. Feeding Trials

Traditionally, animal feeding trials focused on protein quality are performed in growing male rats. In those trials, the parameters used are daily food intake, body weight gain, relative organ weights, net protein utilization (NPU), apparent protein digestibility and biological value. These are well-established parameters based on previous studies in which no difference in the responses of male and female animals was significant [22]. Therefore, in the present study, male Wistar rats were weaned at 21 days of age and given a commercial stock diet until their weights reached 55–60 g. Next, they were fed with the EW diet *ad libitum* for 3 days as a period of adaptation to pulverized diets and were then selected according to food consumption and body weight. The animals were divided into 4 groups of twelve rats each, housed individually in screen-bottomed cages and fed with control (EW), non-protein (NPC) or experimental diets (Seridó-RCH or Seridó) for 10 days. Feed and water were supplied *ad libitum* under adequate sanitary conditions. The rats’ weights, diet spillage and refused diets were recorded daily. Feces were collected during the last 5 days of the experimental period, bulked, freeze dried, weighed and ground in a coffee grinder. At the end of the trial, the rats were euthanized by halothane overdose, and their internal organs were dissected. The contents of the stomachs and intestines were rinsed three times with 0.01 M phosphate buffer solution (PBS) containing 0.15 M NaCl, pH 7.4. The organs were then freeze dried, whereas the carcasses were dried in an oven at 100 °C for 24 h. Dry weights were recorded before incorporating the organs with their original carcasses, which were then ground and kept in a desiccator for appropriate analyses. This study was approved by the Animal Experimentation Ethics Committee of Federal University of Ceará (CEPA) in accordance with the N.11.794/2008 Act, which governs the breeding and use of laboratory animals for teaching and research purposes across the country.

### 2.7. Chemical Analyses

Diets, carcasses and ground fecal samples were analyzed for moisture content and total nitrogen [23,24]. The data were used to calculate apparent protein digestibility (D), NPU and biological value (BV) [22]. The results were calculated for each rat, and the mean within each group was determined.

### 2.8. Statistical Analyses

The results were subjected to a one-way analysis of variance. The significance of differences between two means was determined by Student’s *t* test, and the Tukey honest test was used when comparing multiple means. Multiple regression analysis was applied, relating trypsin inhibitor, lectin, peroxidase, chitinase, β-1,3-glucanase and urease to food intake, weight gain, NPU and digestibility. Due to the high degree of multicollinearity presented in the samples among the independent variables (lectin, trypsin inhibitor, urease, peroxidase, chitinase and β-1,3-glucanase), a technique of orthogonalization was employed [25]. Principal components were constructed, and a special test was performed to discard non-significant components [26].

## 3. Results

### 3.1. Antinutritional and/or Defense Proteins

The activities related to several classical antinutritional and/or defense proteins in the soybean cultivars are presented in Table 2. Trypsin inhibitor activity (g of trypsin inhibited per kg of flour) was significantly higher in the crude extract from the Seridó-RCH cultivar than in that from Seridó. The highest hemagglutinating activity (g of lectin equivalents per kg of flour) measured against rabbit erythrocytes was detected for Seridó-RCH compared with the Seridó cultivar. Regarding urease activity (units of enzyme per kg flour), it was observed that the value for Seridó-RCH was approximately five-fold that for Seridó. Table 2 also shows the activities of proteins related to pathogenesis (PR proteins), such as peroxidase and chitinase. The results showed that seeds from the resistant cultivar Seridó-RCH had higher levels of constitutive peroxidase and chitinase activities compared with seeds from Seridó. The crude extracts of Seridó-RCH and Seridó did not show β-1,3-glucanase activity.

### 3.2. Amino Acid Composition

For all the studied amino acids, no significant difference was observed between Seridó and Seridó-RCH (Table 3). Except for tryptophan, both cultivars contain adequate levels of essential amino acids when compared with the requirements for children [27]. In comparison with requirements for rats, except for lysine and phenylalanine + tyrosine, both cultivars were deficient in all essential amino acids, with tryptophan and methionine + cysteine being the first and second limiting amino acids, respectively. Thus, the diets containing the raw seed flours were supplemented with tryptophan and methionine to eliminate the deficiency of these constituents.

**Table 2:** Biological activities present in the crude extracts of soybean cultivars *.

Activity	Cultivar	
	Seridó	Seridó-RCH
trypsin inhibitor *^a^*	37.50 ± 1.10 a	59.71 ± 1.40 b
Lectin *^b^*	0.10 ± 0.01 a	0.45 ± 0.01 b
Urease *^c^*	132,000 ± 11.32 a	630,000 ± 17.44 b
Peroxidase *^d^*	0.29 ± 0.04 a	3.92 ± 0.01 b
Chitinase *^e^*	17.76 ± 0.90 a	23.50 ± 2.00 b
β-1,3-glucanase *^f^*	ND ^g^	ND

* The values are the mean ± standard deviation of triplicate samples. Values with different letters in the same row differ significantly (*p* < 0.05). *^a^* Trypsin inhibitor activity is expressed as g of trypsin inhibited per kg of flour. *^b^* Lectin activity is expressed as g of lectin equivalents per kg of flour. *^c^* Urease activity is shown as units of enzyme per kg of flour. The units were calculated from information that 1 g of pure enzyme contains 870,000 units, as indicated by Sigma. *^d^* One unit of peroxidase activity was defined as the amount of enzyme that causes an increase of one absorbance unit per minute at 480 nm. *^e^* Chitinase activity was expressed as nanokatal (nkat) per gram of flour (nkat/g) and was defined as the amount of enzyme that releases 1 nmol *N*-acetyl-d-glucosamine per second. *^f^* β-1,3-glucanase activity was calculated as nkat per gram of flour (nkat/g) and was defined as the amount of enzyme catalyzing the formation of 1.0 nm glucose equivalent per second. *^g^* Not detected.

**Table 3:** Comparison of the amino acid composition (g/kg of protein) of the soybean cultivars with WHO/FAO/UNU [27] patterns of amino acid requirements for children and with those required for rats [21] *.

Amino acid	Soybeans		Rat requirement	Requirement for children (3–10 years)
	Seridó	Seridó-RCH		
**Essential**				
Thr	37.0	38.5	40	25
Val	46.8	47.2	55	40
Ile	37.7	39.8	50	31
Leu	76.4	76.1	80	61
Lys	65.8	68.4	60	48
Phe + Tyr	107.2	107.0	90	41
Met + Cys	30.3	30.3	45	24
Trp	7.5	8.8	15	6.6
**Non-essential**				
Asx	116.7	118.8		
Glx	188.4	186.4		
Ser	42.5	41.5		
Gly	38.7	39.3		
Ala	41.2	41.9		
His	29.0	30.3	25	16
Arg	81.5	74.7	50	
Pro	53.1	51.0		

* The values are means of a duplicate. SD values were omitted because they were less than 5% of the means.

### 3.3. Nutritional Parameters

Although daily food intake was not significantly different between the test diets and the EW control, the nutritional parameters were significantly lower for Seridó and Seridó-RCH compared with those calculated for the EW control. The body weight gain of the rats fed with test diets were compared with those of animals fed with EW and NPC diets (Table 4). Both test diets caused body weight gain much lower than that observed for the EW control but were able to promote animal growth, in contrast to the NPC diet. Obviously, this was an expected result because we were comparing a reference animal protein with plant proteins, and specifically legume seed proteins [28]. The Seridó diet presented higher values of NPU (48.2%), protein digestibility (63.8%) and body nitrogen (8.5 g/kg body weight) compared with Seridó-RCH, which showed values of 28.8%, 52.7% and 6.8 g/kg, respectively.

**Table 4:** Nutritional parameters of rats fed on Seridó and Seridó-RCH seed-based defatted flours compared with those of rats fed on egg white (EW) and non-protein control (NPC) diets *.

	Diets			
	NPC	EW	Seridó	Seridó-RCH
initial body weight (g)	71.7 ± 2.8 a	71.8 ± 2.6 a	71.3 ± 2.2 a	71.7 ± 2.4 a
final body weight (g)	56.6 ± 2.9 c	113.4 ± 8.5 a	85.9 ± 7.0 b	81.4 ± 6.4 b
body weight gain (g)	−15.2 ± 1.5 d	42.6 ± 1.7 a	14.4 ± 0.8 b	9.8 ± 1.2 c
daily food intake (g)	2.1 ± 0.6 b	3.7 ± 0.6 a	3.5 ± 0.7 a	3.7 ± 0.7 a
net protein utilization (%)	-	90.7 ± 1.2 a	48.2 ± 1.0 b	28.8 ± 1.0 c
protein digestibility (%)	-	95.4 ± 1.0 a	63.8 ± 1.0 b	52.7 ± 1.6 c
biological value (%)	-	95.1 ± 2.3 a	75.6 ± 0.4 b	54.7± 3.5 c
body nitrogen (g/kg)	8.0 ± 0.8 c	10.3 ± 1.0 a	8.5 ± 0.4 b	6.8 ± 0.4 d

* The values are the mean ± standard deviation (*n* = 12). Values with different letters in the same row differ significantly (*p* < 0.05).

The relative organ weights (g per 100 g of body weight) are presented in Table 5. The diets based on Seridó and Seridó-RCH led to organ weight changes in comparison with the internal organs of EW-fed rats. In general, the experimental diets caused atrophy of the thymus and spleen and enlargement of the small intestine and pancreas based on alterations of their relative dry weights. These changes were even more significant in rats fed on a Seridó-RCH diet. The rats fed on an NPC diet showed divergence in the weights of various organs in relation to the rats fed on EW diet, which can be explained by protein starvation.

**Table 5:** Relative organ dry weights (g/100 g body dry matter) of rats fed with non-protein control (NPC), egg white (EW) and experimental diets *.

Organ	Diets			
	NPC	EW	Seridó	Seridó-RCH
stomach	0.67 ± 0.03 b	0.61 ± 0.04 ac	0.63 ± 0.02 ab	0.58 ± 0.04 c
intestine	2.42 ± 0.44 a	2.25 ± 0.20 a	2.83 ± 0.22 b	3.24 ± 0.13 c
cecum + colon	0.67 ± 0.04 b	0.70 ± 0.04 ab	0.74 ± 0.06 a	0.74 ± 0.06 a
liver	4.52 ± 0.35 b	5.19 ± 0.44 a	4.29 ± 0.25 b	4.57 ± 0.32 b
pancreas	0.28 ± 0.02 a	0.27 ± 0.02 a	0.40 ± 0.04 b	0.51 ± 0.05 c
thymus	0.14 ± 0.01 d	0.28 ± 0.02 a	0.23 ± 0.02 b	0.19 ± 0.01 c
spleen	0.14 ± 0.01 b	0.25 ± 0.01 a	0.16 ± 0.01 b	0.13 ± 0.02 b
kidneys	0.90 ± 0.05 b	0.79 ± 0.06 a	0.75 ± 0.04 a	0.77 ± 0.04 a
heart	0.37 ± 0.04 b	0.29 ± 0.01 a	0.29 ± 0.02 a	0.31 ± 0.02 a
lungs	0.49 ± 0.05 a	0.47 ± 0.03 a	0.46 ± 0.05 a	0.45 ± 0.02 a

* The values are the mean ± standard deviation (*n* = 12). Values with different letters in the same row differ significantly (*p* < 0.05).

### 3.4. Relationships between Unintended Effects and Nutritional Performance

The results of the regression analyses are depicted in Table 6. The technique of orthogonalization used in this study to correct for the high degree of multicollinearity presented by the data only works when collinearity is not perfect. This was not the case for the variable urease. After discarding this variable, principal components were constructed from lectin, trypsin inhibitor, peroxidase and chitinase, and regressions of the nutritional parameters were run on the principal components retained. From these regressions, the coefficients of the original independent variables were recovered. This analysis showed that besides lectin and trypsin inhibitor, peroxidase and chitinase were also significant explanatory variables for the protein quality indicators. In the two cultivars, all of these proteins significantly influenced the food intake in the reverse direction at the same magnitude (−0.60, −0.61, −0.59 and −0.60 were the respective estimated coefficients). Lectin, trypsin inhibitor and chitinase had a significant negative influence on body weight gain, but the effect of the first two proteins was much higher than that of chitinase, as shown by their respective estimated coefficients (−3.33, −3.01 and −1.96, respectively). Regarding NPU, lectin, trypsin inhibitor and chitinase had a negative influence, with estimated coefficients of −2.55, −0.62 and −4.81, respectively. Additionally, lectin and trypsin inhibitor had a negative effect on digestibility (estimated coefficients of −8.43 and −5.22, respectively), whereas chitinase had a positive impact (estimated coefficient of 3.99). Peroxidase did not show any significant effect on body weight gain, NPU or digestibility.

However, in the case of organ weights, the variables urease, lectin, trypsin inhibitor, peroxidase and chitinase presented a degree of collinearity, which prevented any regression analysis, even with orthogonalization techniques. Therefore, regression analysis is not presented to explain the organ weights.

**Table 6:** Regression results: estimated coefficients, T-ratios and *R*^2^.

Independent variable	Dependent variables			
	Diet Intake	Weight Gain	NPU	Digestibility
lectin	−0.60 (−6.45) *	−3.33 (−4.27) *	−2.55 (−36.76) *	−8.43 (−8.26) *
trypsin inhibitor	−0.61 (−6.45) *	−3.01 (−4.63) *	−0.62 (−18.25) *	−5.22 (−10.41) *
peroxidase	−0.59 (−6.45) *	2.86 (1.69)	−0.04 (−0.48)	0.14 (0.13)
chitinase	−0.60 (−6.45) *	−1.96 (−7.79) *	−4.81 (−47.88) *	3.99 (2.7) *
*R*^2^	0.81	0.93	0.99	0.98

* Significant at α = 0.05.

## 4. Discussion

The results showed that the resistant cultivar obtained by conventional breeding did not have its amino acid profile altered, although there were changes in the activities of certain antinutritional and/or defense-related proteins. Indeed, the activities of all the analyzed proteins were higher in the more resistant (Seridó-RCH) soybean cultivar, except for β-1,3-glucanase, whose activity was not detected in either cultivar. The trypsin inhibitor content in the seed flour of Seridó-RCH was nearly 63% higher than that in the Seridó cultivar. Trypsin inhibitors are proteins widely distributed in plant species. Their roles are diverse, including one of the most important defense strategies used by plants to combat phytophagous insects and microorganisms [29,30]. It is possible that the trypsin inhibitor of soybean belongs to the arsenal of molecules related to the higher resistance of Seridó-RCH against fungi. The action of certain protease inhibitors against bacteria has also been reported [31]. Nevertheless, to the knowledge of the authors, no association of soybean trypsin inhibitors with activity against bacteria has been described so far. Regarding lectins, they belong to a class of protein also related to plant defense against insects, fungi, bacteria, and nematodes [32,33]. Although many plant lectins are toxic to phytopathogenic fungi and bacteria, there are no reports specifically on soybean lectins with these activities. Therefore, the resistance of soybean to these organisms is apparently not related to this class of protein. However, the Seridó-RCH cultivar presented higher levels of lectin compared with the Seridó cultivar in the present study. This is perhaps an example of unintended effects obtained after the process of conventional breeding.

Ureases are metalloenzymes present in virtually all plant species and ubiquitously distributed in plant tissues [34]. An embryo-specific soybean urease has been shown to impair growth of selected phytopathogenic fungi at sub-micromolar concentrations [35]. Moreover, it has been shown that soybean plants with no urease were more susceptible to fungi than plants containing normal levels of the enzyme [36]. Thus, our data indicate that urease may be involved in the resistance of Seridó-RCH to fungi.

Regarding peroxidase, chitinase and β-1,3-glucanase, these proteins are related to defense (PR proteins) and can be present constitutively in plants and are therefore involved in resistance to herbivores and pathogens [37]. The levels of these proteins were analyzed in the seed flour of both cultivars in the current study. Peroxidase, for example, presented a specific activity 14 times higher in the Seridó-RCH cultivar than in the Seridó cultivar. This enzyme is involved in plant defense against several stress agents, and its level can be associated with higher or lesser plant resistance [38]. There are also reports of peroxidase with direct toxicity against certain species of fungi and bacteria [39]. Seridó-RCH also presented higher chitinase activity compared with Seridó. The natural substrate of chitinases, chitin, is often present in fungal hyphae as the main component of the cell wall [40]. The toxicity of chitinases against fungi has been widely described [41]. However, data related to the direct action of these proteins against bacteria are rare. However, certain plant chitinases with additional lysozyme or lysozyme-like activity are able to cleave bacterial peptidoglycan [42]. In the present work, it was observed that conventional breeding of soybean cultivars produced a coordinated increase in several proteins involved in plant defense, which caused higher resistance in the Seridó-RCH cultivar. Efforts to engineer disease resistance in plants through the overexpression of PR proteins have shown that these proteins are not as effective when induced individually compared with when they are coordinately expressed [43].

Little attention has been paid to the possible food safety of new plant varieties derived from conventional breeding. However, the potential risks of the introduction or increased production of antinutritional or toxic compounds are applicable to the development of crop cultivars using both conventional breeding methods and transgenic plant technology [44]. To characterize the likelihood of unintended effects of the resistant cultivar (Seridó-RCH), with subsequent deleterious effects on humans and/or animals, a feeding trial was conducted in rats, using both cultivars as the sole source of protein. Diets were prepared using seeds produced under strict quality standards that were apparently free of pathologies related to microbial contamination and aflatoxins. Moreover, soybean seeds were not previously processed by autoclaving and/or irradiation, for example, to avoid changes, particularly in native protein constituents such as lectins, protease inhibitors and other proteins. Several studies have shown that autoclaving and irradiation alter the nutritional quality of certain proteins. Gamma ray exposition decreased the protein content of certain edible seeds in a dose-dependent manner, increasing the amount of free amino acids. In addition, this treatment decreased protein solubility, as observed for the albumin and globulin fractions of certain oil seeds (soybean, peanut and sesame) [45,46]. Furthermore, it is possible that radiation causes protein fragmentation and aggregation, with the latter effect leading to protein insolubility [47,48]. A protein-free (NPC) diet and an EW-based diet were used as controls. Although the daily food intake and amino acid composition were similar between the two cultivars, the rats fed with Seridó-RCH diets generally showed worse performance when protein quality indicators were compared.

A regression analysis relating the antinutritional and/or defense proteins to the nutritional parameters showed that lectin, trypsin inhibitor, peroxidase and chitinase were significant explanatory variables for the protein quality indicators. Lectin, trypsin inhibitor, peroxidase and chitinase significantly influence food intake in the reverse direction. However, weight gain is inversely influenced by lectin, trypsin inhibitor and chitinase, whereas peroxidase has no significant influence. NPU is also inversely influenced by lectin, trypsin inhibitor and chitinase. However, digestibility is significantly and inversely influenced by lectin and trypsin inhibitor but directly affected by chitinase. The antinutritional action of trypsin inhibitors and lectins is well known. Trypsin inhibitors decrease the availability of protein needed for optimal growth and health because they reduce the intestinal ability to digest protein, whereas lectins are known for their specific binding to cellular and intracellular membrane-associated carbohydrates (glycoprotein and glycolipids). Lectins also bind to carbohydrates in ingested food and limit or change their potential hydrolysis and absorption from the gut [49,50]. Regarding chitinase, the results showed that it contributed negatively to body weight gain as well as to NPU but not to digestibility. This phenomenon might have contributed to a lower biological value for the Seridó-RCH cultivar because this parameter may be calculated by dividing NPU by digestibility. These results, showing that chitinase is an antinutrient, are relatively surprising because nothing has ever been reported in this sense. Thus, further studies must be conducted to clarify this concept.

Although it was not possible to apply multiple regression analysis to the effects of the different proteins on organ weights, the diet based on Seridó-RCH, which exhibited higher activities for all of the studied proteins compared with Seridó, caused more prominent changes in certain organs, such as thymus atrophy and small intestine and pancreas enlargement. In previous work with other soybean cultivars, regression analysis data showed that small intestine enlargement was mainly induced by lectin, although trypsin inhibitor also had a significant effect. Conversely, trypsin inhibitor was the main factor responsible for pancreas enlargement, although lectin was also a significant explanatory variable. In the same study, urease was also a significant explanatory variable for pancreas and small intestine enlargement [4]. Thus, in the present study, it is probable that lectin, trypsin inhibitor and urease also contributed to alterations in these key organs.

## 5. Conclusions

Although they have the same protein contents, the non-isogenic soybean cultivars Seridó and Seridó-RCH, which are susceptible and resistant to stem canker, respectively, differed significantly in their levels of antinutritional and plant defense proteins. The increase in these proteins verified for Seridó-RCH was experimentally shown to cause a reduction in the protein quality of this resistant soybean cultivar. Although we cannot generalize our conclusion, the purpose of this work was to raise concerns about the possibility that conventional breeding processes for developing disease-resistant cultivars may bring about undesirable traits of this nature.
